# Case Report: Fatal mesenteric venous thrombosis and progressive bowel ischemia following right hemihepatectomy

**DOI:** 10.3389/fsurg.2026.1830475

**Published:** 2026-05-19

**Authors:** Zhun Shen Tan, Elvin Jia Hong Lee, Rou Bei Tan, Azlanudin Azman, Ian Chik

**Affiliations:** 1 Faculty of Medicine, National University of Malaysia, Kuala Lumpur, Malaysia; 2 Hepatobiliary & Pancreatic Surgery Unit, Department of Surgery, Faculty of Medicine, National University of Malaysia, Kuala Lumpur, Malaysia

**Keywords:** bowel ischemia, case report, hepatectomy, klatskin tumor, mesenteric ischemia, mesenteric venous thrombosis, postoperative complications, Virchow's triad

## Abstract

Mesenteric venous thrombosis (MVT) is an uncommon but potentially fatal complication following major hepatic resection. We report the case of a 62-year-old cirrhotic male with Hepatitis B who underwent an open right hemihepatectomy with Roux-en-Y reconstruction for a suspected Bismuth-Corlette Type 3A Klatskin tumour. On postoperative day (POD) 4, he developed refractory metabolic acidosis with profound hyperlactatemia, prompting urgent CT imaging and exploratory laparotomy which revealed extensive patchy small bowel ischaemia consistent with MVT. Serial laparotomies demonstrated relentless progression to total intestinal infarction despite maximal surgical and intensive care intervention and the patient succumbed to septic shock and multi-organ failure on POD 14. This case illustrates how the convergence of Virchow's triad in the post-hepatectomy cirrhotic patient, namely portal stasis exacerbated by inadequate fluid resuscitation, cirrhosis-mediated hypercoagulability and surgical endothelial injury can precipitate a rapidly fatal thrombotic cascade. It further highlights the dual clinical dilemma of diagnostic elusiveness as early imaging may be falsely negative and therapeutic paralysis where the perceived haemorrhagic risk of anticoagulation in a coagulopathic patient may delay the only intervention capable of halting thrombus propagation. This case underscores the imperative for heightened clinical vigilance, proactive hydration and a low threshold for repeat vascular imaging in cirrhotic patients undergoing major hepatectomy. Early anticoagulation tailored to the individual patient's risk profile should be carefully considered upon clinical suspicion of MVT, even in the setting of apparent coagulopathy.

## 1 Introduction

Right hemihepatectomy is the established curative procedure for Klatskin tumors with predominant right-sided extension (1). The postoperative course is commonly complicated by liver failure, biliary leak, and hemorrhage (1). While thrombotic events are recognized, they most frequently involve the portal vein (2, 3). Mesenteric venous thrombosis (MVT) is an uncommon yet potentially lethal complication of major hepatic resection, presenting with a broad clinical spectrum that often includes nonspecific early symptoms, resulting in delayed diagnosis (4, 5).

This risk is significantly elevated in the setting of cirrhosis, a condition associated with a baseline hypercoagulable state (2). The procedure itself alters portal venous hemodynamics, which, in a patient with underlying coagulopathic predisposition, may precipitate thrombosis within the mesenteric venous system (4). Management presents a lethal paradox where the disease mandates immediate anticoagulation, yet the postoperative state may preclude it, resulting in the rapid and fatal bowel infarction exemplified in this case (4, 6). High-level evidence-based protocols are lacking due to the absence of randomised trials, a consequence of the condition being uncommon, often asymptomatic, and frequently overlooked in the postoperative setting (6).

This report details the clinical course of a fatal MVT after right hemihepatectomy in a cirrhotic patient. We chronologically analyze the case to illustrate the diagnostic and therapeutic dilemmas it presents, framing its pathogenesis through the lens of Virchow's triad to contextualize this devastating complication.

## 2 Case report

### 2.1 Patient presentation and preoperative optimization

A 62-year-old gentleman with no significant prior medical history presented with a two-week history of painless jaundice and epigastric discomfort. Serology confirmed a new diagnosis of chronic Hepatitis B virus infection (HBsAg reactive, HBV DNA 5,430 IU/mL).

**Figure 1:**
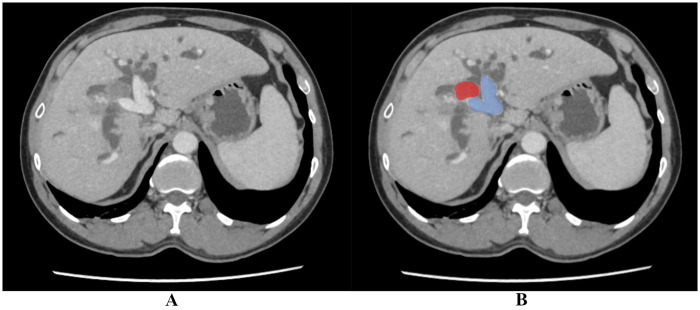
Preoperative MRI findings demonstrating the bismuth-corlette type 3A klatskin tumour. **(A)** Unannotated MRI showing a heterogeneous hilar mass at the confluence of the common hepatic duct with a patent main portal vein. **(B)** Annotated MRI with the tumour highlighted in red and the patent portal vein in blue.

Cross-sectional imaging revealed a 2.4 × 4.2 × 4 cm heterogenous mass at the confluence of the common hepatic duct, consistent with a Bismuth-Corlette Type 3A Klatskin tumor, with associated bilateral intrahepatic ductal dilation with a patent main portal vein (Figure 1). Preoperative serum CA 19-9 was elevated at 102 U/mL.

Persistent hyperbilirubinemia (114.60 μmol/L) due to obstruction was noted. Following endoscopic biliary stenting and percutaneous transhepatic biliary drainage (PTBD), his liver function improved. Calculated future liver remnant (FLR) was 43%. After a multidisciplinary tumor board discussion, he was scheduled for curative-intent surgical resection.

### 2.2 Surgical management and clinical progression

**Figure 2:**
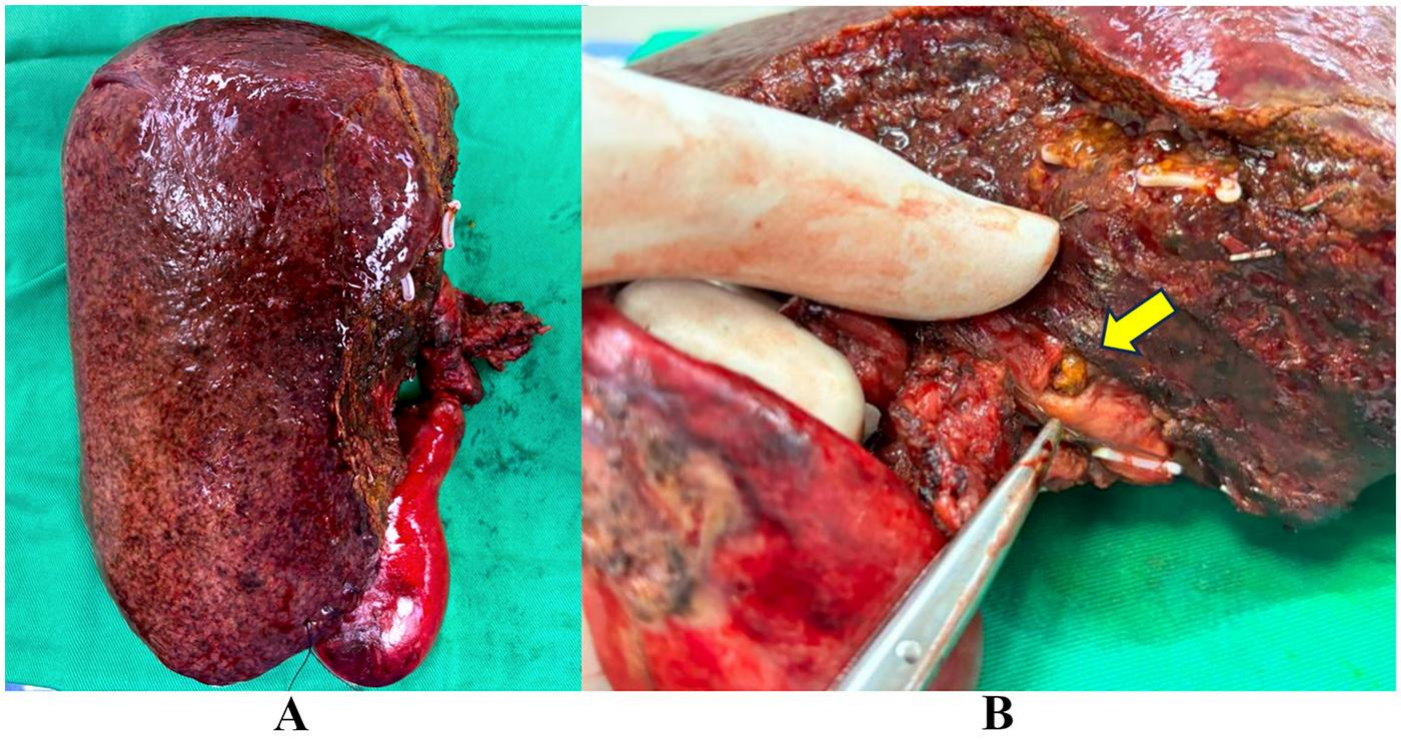
Resected surgical specimen following open right hemihepatectomy. **(A)** Gross specimen comprising the right hepatic lobe and gallbladder. **(B)** Klatskin tumour identified at the hepatic hilum (yellow arrow), demonstrating its location at the right intrahepatic duct.

The patient underwent an open right hemihepatectomy with partial caudate lobectomy and Roux-en-Y hepaticojejunostomy on 16 December 2025. Intraoperative findings confirmed a cirrhotic liver. The right portal vein was ligated and divided, parenchymal transection was performed under an intermittent Pringle maneuver (four cycles of 15 min; total ischaemic time 60 min), and a double-duct hepaticojejunostomy was fashioned (Figure 2). The immediate postoperative period in a high-dependency unit was initially stable. Prophylactic low-molecular-weight heparin was withheld postoperatively in view of the persistently prolonged INR, following multidisciplinary discussion regarding haemorrhagic risk in the immediate post-hepatectomy period.

**Figure 3:**
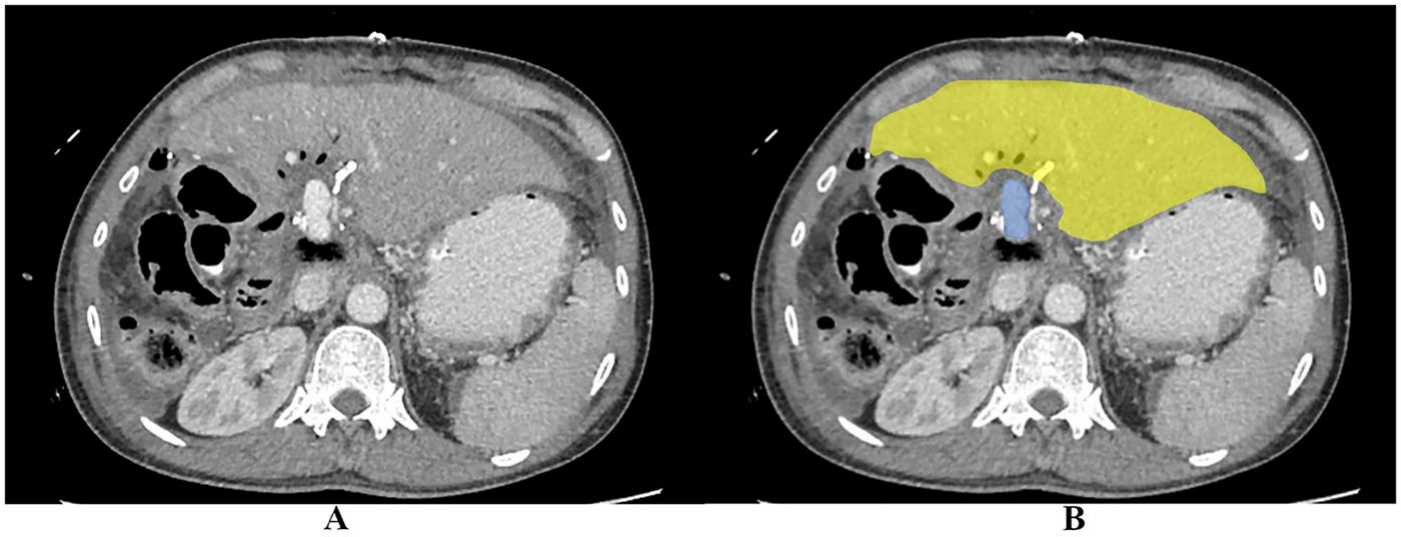
Preoperative CT obtained during the period of bowel ischaemia on POD 4. **(A)** Unannotated CT demonstrating a patent portal vein without evidence of portal vein thrombosis. **(B)** Annotated CT with the patent portal vein highlighted in blue and the residual liver remnant in yellow, confirming portal patency in the context of mesenteric venous thrombosis.

**Figure 4:**
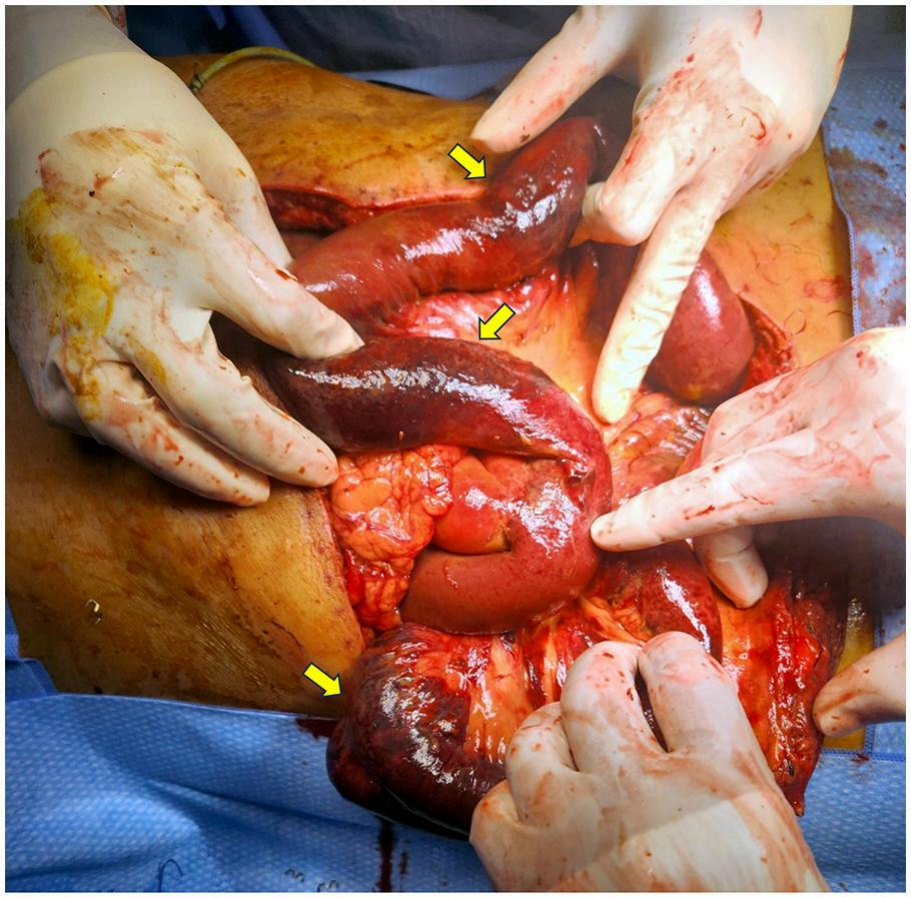
Intraoperative findings at initial exploratory laparotomy on POD 4, demonstrating patchy, segmental ischaemia of the small bowel (yellow arrows), consistent with the diagnosis of MVT.

On postoperative day (POD) 2, the patient demonstrated persistent hyperlactatemia with metabolic acidosis and tachycardia, although he remained clinically stable without vasopressor requirement. A dramatic clinical decline on POD 4 prompted the first emergency intervention. There was worsening metabolic acidosis, new-onset abdominal discomfort, escalating tachycardia and the need for low-dose vasopressor support, ultimately necessitating intubation for tachypnoea. An urgent CT abdomen was performed with the portal vein noted to be patent on imaging, excluding portal vein thrombosis as the primary aetiology and pointing toward isolated MVT as the underlying cause (Figure 3). However, no mesenteric venous thrombosis was identified on imaging as well. An exploratory laparotomy that same day revealed 1 L of serous fluid upon entering the peritoneum and approximately 120 cm of patchy ischaemic small bowel, with the remaining proximal bowel healthy to 100 cm from the duodenojejunal junction and the distal bowel healthy to 80 cm from the ileocaecal valve. The macroscopic appearance was consistent with MVT which was diagnosed intraoperatively (Figure 4). The hepaticojejunostomy anastomosis remained intact. No intestinal anastomosis was fashioned at this stage where a laparostomy was instead created to allow planned reassessment of bowel viability at second-look laparotomy.

The laparostomy wound was re-explored the next day (POD 5). This procedure revealed progressive ischemia, necessitating further resection of 10 cm of proximal jejunum. The distal bowel remained viable to 80 cm from the ileocaecal valve, upon which a definitive double-barrel ileostomy was fashioned.

The subsequent week (POD 5-12), the patient stabilised with improving lactate and acidotic levels. During this interim as thrombus propagation remained a clinical concern, a formal multidisciplinary discussion involving the intensivist, haematologist, gastroenterologist and surgeon was reconvened to reassess the role of therapeutic anticoagulation. While the surgeon advocated for its initiation, the gastroenterologist and haematologist remained opposed, citing the persistently prolonged INR ranging from 1.57 to 2.02 as representing a prohibitive haemorrhagic risk in the setting of ongoing liver failure. Consensus could not be reached and anticoagulation was again deferred. In retrospect, a more comprehensive appreciation of the rebalanced haemostatic state in cirrhosis may have supported earlier initiation of anticoagulation with close clinical monitoring. However on POD 13, there was a sudden deterioration. The patient developed progressive septic shock requiring escalating vasopressor support.

A final, definitive laparotomy revealed the catastrophic extent of the disease. Findings included extensive necrosis of the remaining small bowel and large colon with a perforation at the jejunojejunal anastomosis secondary to ischemic necrosis. Given the non-viability of the remaining intestinal tract, further resection would have conferred no clinical benefit. The procedure was therefore limited to peritoneal washout and drainage as a palliative measure before abdominal closure.

### 2.3 Outcome

Despite maximal intensive care, including mechanical ventilation, escalating vasopressors, targeted antibiotics, and liver failure management, his condition deteriorated into worsening septic shock with acute kidney injury and coagulopathy. The extensive and progressive bowel necrosis resulted in uncontrolled intra-abdominal sepsis, leading to refractory lactic acidosis, cardiovascular collapse, and ultimately asystolic cardiac arrest on the morning of POD 14. In accordance with family wishes, a do-not-resuscitate order was in effect and the patient was pronounced deceased following the arrest.

The final clinical cause of death was severe intra-abdominal sepsis with multi-organ failure following right hemihepatectomy for Klatskin tumor, complicated by mesenteric venous thrombosis and catastrophic bowel infarction.

### 2.4 Patient perspective

Informed written consent for the publication of this case report was obtained from the patient's next of kin. The family expressed that they hoped the documentation and analysis of this tragic clinical course might contribute to medical understanding and aid in the future recognition and management of this rare complication for other patients and families.

## 3 Discussion

This case provides a stark illustration of a rare and almost uniformly fatal complication following major hepatectomy. The catastrophic progression from surgery to total intestinal infarction within two weeks underscores a critical vulnerability in postoperative care for cirrhotic patients. The pathophysiology is not straightforward, the diagnosis is elusive and the management lacks clear guidance.

### 3.1 Pathophysiological analysis: A clinical demonstration of Virchow’s triad

The catastrophic progression of MVT in this patient can be decisively mapped to the three elements of Virchow's triad, each uniquely amplified by the specific clinical context of major hepatectomy in cirrhosis.

The first element of the triad, stasis, was iatrogenically established by the index procedure (6). Right hemihepatectomy involves the resection of the larger volume of the liver and leaving a relatively small liver remnant, often leading to higher rate of postoperative mortality (1). Major resections of this nature with the ligation of the right portal vein represent a critical hemodynamic insult that acutely disrupts portal flow (4). The resultant decreased portal venous flow creates the stasis required for fibrin formation within the superior mesenteric vein (7). In the acute post-operative state, this condition is defined by the absence of porto-portal collaterals, as collateral circulation has not yet developed to compensate for the obstruction (4). This stasis of the portal blood flow directly satisfies Virchow's triad regarding the alteration in blood flow, providing the necessary environment for thrombogenesis (7). Further amplifying this risk, mesenteric venous blood is highly susceptible to fluid shifts and changes in viscosity, which can occur during periods of dehydration or fasting (2). This contributes towards the stasis required for fibrin formation and secondary haemostasis (7).

The second element, a hypercoagulable state, was inherent to the patient's cirrhosis (2). While laboratory derangements such as elevated prothrombin time/international normalized ratio (PT/INR), partial thromboplastin time (PTT) or thrombocytopenia may suggest a bleeding risk, patients with liver insufficiency actually have a significantly increased risk for venous thrombosis (4). This state is mechanistically driven by a systemic reduction in natural anticoagulants, specifically antithrombin III, protein C, and protein S (2, 4). Major surgery acts as a potent catalyst by inducing acquired prothrombotic states, such as sepsis or inflammatory responses, which elevate tissue factor expression and push this fragile equilibrium into clinical thrombosis (4, 7). Furthermore, the creation of a small liver remnant during major resection correlates with an increased von Willebrand factor ratio, which directly enhances thrombogenesis (4).

The third and final component, endothelial injury, is an inevitable consequence of the operative trauma inherent to major hepatic surgery (4, 6). Hepatectomy involves extensive intraoperative vessel manipulation, ligation of major portal tributaries, and, in selected cases, venous resection or reconstruction, all of which result in direct injury to the vascular endothelium (4). Furthermore, the use of the Pringle maneuver (clamping of the hepatoduodenal ligament) is frequently required during hepatectomy, contributing to portal vein endothelial injury through ischemic and mechanical stress (4). This endothelial disruption exposes subendothelial tissue and collagen to circulating blood, initiating platelet adhesion and triggering tissue factor synthesis, which activates the coagulation cascade (7).

### 3.2 The clinical dilemma: diagnostic uncertainty and therapeutic risk

This case illustrates an exceptionally challenging clinical scenario, primarily due to the diagnostic difficulty associated with acute MVT. Early clinical manifestations are often nonspecific and variable, commonly presenting as abdominal pain, nausea, and vomiting (4). Diagnostic evaluation is further complicated by the absence of a reliable plasma biomarker as serum lactate elevates only once ischaemia is advanced and irreversible, rendering it inadequate for early detection (4, 6). Consequently, these symptoms are frequently misattributed to more common postoperative sequelae, such as post-hepatectomy liver failure (PHLF) following major hepatic resection (4, 8). In the absence of overt clinical concern, routine imaging is often deferred, leading to delayed diagnosis until irreversible or chronic changes have developed (4). In this patient, contrast-enhanced computed tomography (CECT) performed on POD 2 for persistent acidosis revealed no evidence of portal vein thrombosis or mesenteric ischemia. MVT was only diagnosed intraoperatively based on characteristic findings of patchy bowel ischaemia, underscoring the diagnostic elusiveness of early MVT.

This diagnostic challenge is partly attributable to the fact that while CECT is highly sensitive, its accuracy is exclusively dependent on imaging strictly within the portal venous phase to ensure adequate opacification of the mesenteric venous system (4, 6). Sensitivity for identifying the thrombus itself is high, yet the inter-reader agreement for secondary intestinal abnormalities is slightly lower (6). Early CT findings such as bowel wall thickening, mesenteric oedema, and ascites are frequently observed but are often non-specific as they can also be present in other conditions like postoperative pancreatitis or haemorrhage (4, 5). Consequently in the absence of a clearly visible intraluminal filling defect, these findings may only strongly suggest the diagnosis, potentially rendering an early, definitive diagnosis difficult (4, 5).

Therefore, maintaining a high index of suspicion is essential, particularly when leukocytosis is accompanied by metabolic acidosis and haemodynamic instability, as these findings should prompt immediate diagnostic imaging (4). Current literature suggests that screening high-risk patients with CECT performed in the portal venous phase on POD 7 may facilitate earlier detection and accurate delineation of thrombus formation (4).

Second, the therapeutic dilemma is profound. The disease pathophysiology mandates immediate anticoagulation with heparin to prevent thrombus propagation (6), as the risk of complete vascular occlusion is considered to far outweigh the risk of bleeding (4). However, the postoperative state presents a prohibitive conflict across both fluid management and anticoagulation decision-making. In terms of fluid management, withholding fluids allows stasis and viscosity to propagate the thrombus and worsens bowel ischemia (2, 4), yet aggressive fluid resuscitation risks triggering catastrophic fluid overload and respiratory or renal failure in a patient whose liver remnant cannot effectively manage the volume (4, 8). In terms of anticoagulation, surgeons frequently delay or withhold anticoagulation following major hepatectomy due to concerns that patients with postoperative liver insufficiency are already “anticoagulated” as evidenced by elevated PT/INR and thrombocytopenia, thereby creating a substantial perceived risk of major haemorrhage (4). Despite derangements in PT/INR, PTT, and platelet count, patients with liver insufficiency paradoxically carry a significantly increased risk for venous thrombosis, as routine coagulation assays fail to capture the concurrent reduction in natural anticoagulants such as protein C, protein S, and antithrombin (4, 9). This is further supported by a prospective cirrhosis cohort of 260 patients, which confirmed that standard coagulation markers are unreliable predictors of bleeding risk as they do not reflect the underlying hypercoagulable state present in cirrhosis (9). In this case following the first episode of mesenteric ischemia, initiation of anticoagulation was warranted to prevent further propagation. Yet, consensus could not be reached given the competing concern of haemorrhagic risk in the setting of ongoing liver failure. Additionally, transvenous thrombolysis or thrombectomy were not pursued given no radiologically identifiable thrombus was detected on imaging to serve as an anatomical target to guide such intervention.

### 3.3 Implications for clinical practice

While portal vein thrombosis is a well-documented post-hepatectomy complication (4), isolated acute MVT is described as uncommon, often overlooked, and literature on the subject is limited to scattered reports consisting of case reports or very small series (4, 10). This rarity explains why high-level evidence is lacking and why optimal management remains uncertain (6, 10). The key lessons from such cases argue for a shift in postoperative vigilance, particularly as symptoms are often unspecific and variable (4).

Firstly, cirrhotic patients undergoing major hepatectomy constitute a distinct high-risk cohort as liver insufficiency and a small liver remnant volume enhance thrombogenesis (2, 4). In this context, a higher functional liver remnant volume is likely required when there is any preoperative indication of cirrhosis, both to reduce the risk of post-hepatectomy liver failure and to mitigate the thrombogenic consequences of an inadequate remnant (4, 8). Postoperatively, maintaining adequate hydration is a critical and modifiable intervention. Given that mesenteric venous blood is highly susceptible to fluid shifts and that dehydration increases viscosity and promotes stasis (2), diligent fluid management represents a direct means of disrupting one arm of Virchow's triad before thrombosis becomes established (7). While precise quantitative fluid balance data were unavailable in this case, clinical features in the early postoperative period were consistent with subclinical intravascular volume depletion (2, 4). This is clinically significant as early postoperative patients routinely sustain substantial third-space losses such that recorded fluid input may inadequately reflect true effective circulating volume (4). This directly increases mesenteric blood viscosity and promotes stasis, thereby reinforcing the proposed pathophysiological mechanism (2).

Furthermore, given the profound and conflicting risks of major haemorrhage vs. thrombus propagation, management requires multidisciplinary preparedness. Pre-emptive consultation with specialists in haematology is essential to define the best therapy, and severe cases involving multi-system failure mandate management within the intensive care unit (4, 8).

This case illustrates the potential natural history when intervention is withheld, characterised by relentless progression toward life-threatening bowel infarction and massive gastrointestinal fluid sequestration (4). Despite these stakes, high-quality comparative studies and randomised trials remain lacking. Based on this clinical course, several actionable recommendations can be drawn. Cirrhotic patients undergoing major hepatectomy should be prospectively identified as a high-risk cohort for thrombotic complications, with thromboprophylaxis protocols reconsidered rather than withheld solely on the basis of a prolonged INR. Close monitoring should then be implemented for bleeding complications. Postoperative hydration should be guided by haemodynamic parameters with vigilance for intravascular depletion despite apparently adequate fluid input. Finally, any unexplained postoperative deterioration should prompt repeat CECT in the portal venous phase rather than attribution to post-hepatectomy liver failure alone. In the absence of evidence-based protocols, management must be guided by the individual clinical context and the available institutional expertise (4, 6).

### 3.4 Study limitations

This case report has several limitations. First, as a single case report the findings cannot be generalised and causality cannot be established. Second, preoperative and serial postoperative measurement of natural anticoagulants including protein C, protein S and antithrombin were not performed which would have better characterised the patient's true prothrombotic state beyond conventional INR and PTT measurements alone. Third, the decision to withhold anticoagulation while clinically understandable given the competing haemorrhagic risk means it remains impossible to determine whether earlier initiation would have altered the outcome. Finally, given the rarity of post-hepatectomy MVT, high-quality comparative data and randomised trials are lacking where management recommendations remain necessarily case-based rather than evidence-based.

## 4 Conclusion

This case illustrates the fatal potential of mesenteric venous thrombosis, a rare but devastating complication following major hepatectomy in a cirrhotic patient. Adequate clinical assessment including vigilant monitoring of hydration status and fluid balance remains paramount in the postoperative period, as inadequate volume resuscitation likely contributed to the portal stasis that precipitated MVT in this case. While mesenteric thrombosis post-hepatectomy is an uncommon clinical scenario, it must be considered whenever a patient is not improving and remains persistently acidotic, particularly in the setting of background liver cirrhosis where the threshold for suspicion must be lower. The clinical course underscores a critical dilemma where diagnostic delay due to nonspecific symptoms conflicts with the prohibitive perceived risk of postoperative anticoagulation. Yet this case argues that in patients with known INR derangement and cirrhosis-mediated coagulopathy, a patient-tailored anticoagulation protocol should be considered early, even in the absence of standardised guidelines, rather than withheld on the basis of conventional bleeding risk parameters alone. This outcome highlights the imperative for heightened clinical vigilance, proactive hydration, and early imaging in high-risk patients to identify a narrowing window for intervention before catastrophic bowel infarction occurs.

## Data Availability

The original contributions presented in the study are included in the article/Supplementary Material, further inquiries can be directed to the corresponding author.
